# Convention of Medical Schools

**Published:** 1850-03

**Authors:** 


*Convention of Medical Schools.*—The American Medical Association at its last meeting adopted the following Resolution:—

"*Resolved,* That the Association recommend to the various schools of medicine to meet at Cincinnati before the next annual meeting of this Association and present a plan for elevating the standard of medical Education to this Association."

As many advantages might accrue from such a convention prior to the meeting of the Association, we hope the recommendation of the Association will be adopted. Should not the Schools enter into some correspondence for this end, and will not some one of the older institutions take the initiatory steps?

